# Causal effect of body mass index and physical activity on the risk of joint sports injuries: Mendelian randomization analysis in the European population

**DOI:** 10.1186/s13018-023-04172-y

**Published:** 2023-09-12

**Authors:** Wen Bi, Mengyue Yang, Changqing Jiang

**Affiliations:** 1 Department of Sports Medicine, Huazhong University of Science and Technology Union Shenzhen Hospital, The 6th Affiliated Hospital of Shenzhen University Health Science Center, Shenzhen, Guangdong China; 2 Department of Cardiology, Huazhong University of Science and Technology Union Shenzhen Hospital, The 6th Affiliated Hospital of Shenzhen University Health Science Center, Shenzhen, Guangdong China

**Keywords:** Mendelian randomization, Body mass index, Physical activity, Joint sports injuries, FinnGen, Causal relationship, Single-nucleotide polymorphisms

## Abstract

**Background:**

Observational studies can suggest potential associations between variables but cannot establish a causal effect on their own. This study explored the causal associations between body mass index (BMI), physical activity (PA), and joint sports injuries.

**Methods:**

We conducted two-sample Mendelian randomization (MR) using publicly accessed genome-wide association studies (GWAS) datasets to investigate the causal effects of BMI and PA on joint sports injury risk. The inverse-variance weighted method was believed to be the primary MR analysis. Subsequently, sensitivity, pleiotropy, and heterogeneity analyses were employed to estimate the reliability of the results of the current research.

**Results:**

Genetically predicted increased BMI was causally related to the higher sports injury risk of the ankle–foot (OR 1.23, 95% CI 1.09–1.37, *p* = 4.20E−04), knee (OR 1.32, 95% CI 1.21–1.43, *p* = 1.57E−11), and shoulder (OR 1.23, 95% CI 1.08–1.40, *p* = 1.28E−03). Further, the mentioned effects were validated using another set of GWAS data on BMI. Similar causal linkages were exhibited between increased BMI and the growing risk of sports injuries of the ankle–foot (OR 1.34, 95% CI 1.13–1.60, *p* = 9.51E−04), knee (OR 1.26, 95% CI 1.09–1.45, *p* = 1.63E−03), and shoulder (OR 1.35, 95% CI 1.09–1.67, *p* = 5.66E−03). Additionally, accelerometer-based PA measurement (overall average acceleration) (AccAve) was negatively related to sports injuries of the ankle–foot (OR 0.93, 95% CI 0.87–0.99, *p* = 0.046) and lumbar spine (OR 0.68, 95% CI 0.51–0.92, *p* = 0.012). Furthermore, we verified that the effect of AccAve on the risk of injury at the ankle–foot still had statistical significance after adjusting BMI. Results were verified as reliable under all sensitive analyses.

**Conclusions:**

This research determined that a higher BMI could raise the sports injury risk of the ankle–foot, knee, and shoulder, while an overall average acceleration PA could reduce the injury risk of the ankle–foot and lumbar spine. These conclusions contribute to a greater knowledge of the roles of BMI and PA in the mechanism of joint sports injuries and offer several suggestions for patients and clinicians.

**Supplementary Information:**

The online version contains supplementary material available at 10.1186/s13018-023-04172-y.

## Introduction

People all around the world love doing sports as hobbies, exercises, and ways to stay healthy. However, compared to transportation-related injuries, home and recreational accidents, and work-related damages, sports are among the leading causes of joint injuries [1–3]. Minor sports-related joint injuries, including dislocation, sprain, and strain, are the most commonly reported [4]. Some factors, including intrinsic factors like BMI, age, and gender, and extrinsic risk factors, such as the type of sport practiced and physical activity, could affect the risk of a joint injury [5]. Determining the risk factors for sports-related joint injuries might assist patients and caregivers in better understanding the etiology and developing care and treatment recommendations.

Body mass index (BMI), which serves as a surrogate indicator for obesity, has been a remarkable risk factor for sports injuries of the joints, such as ankle sprains and strains. Observational studies have reported that an increased BMI is correlated with a greater risk of sports injury [6–9]. Despite this, it should be recognized that the constraints of conventional study approaches, notably underlying confounders or reverse causalities, prevented rigorous confirmation of the correlations [10].

Mendelian randomization (MR) analyses can be an ideal strategy to address these constraints [11]. This method takes advantage of instrumental variants (IVs) as proxies for exposures (e.g., disease, lifestyle), which can be conducive to overcoming the constraints of observational studies. Therefore, MR analyses are an effective approach for enhancing causal inference. PA is an essential factor that should not be ignored in joint sports injuries [5]. The occurrence rate of sports injuries to the ankle varied depending on the intensity of PA [12–14]. Nevertheless, the role of BMI and PA in sports injuries of other joints and whether PA mediates the correlation between BMI and joint injury have not been fully illustrated.

To address this issue, we first performed a two-sample Mendelian randomization analysis to explore the causal effect of BMI and PA on the risk of 13 different types of joint injury in sports. Then, multivariate MR was implemented to verify the causal effect of physical activity on the susceptibility to joint injury, adjusting for potential pleiotropy. With robust IVs, the MR method is less vulnerable to confounders and reverse causalities, which could interfere with the findings compared to conventional research.

## Methods

### Study design

**Fig. 1:**
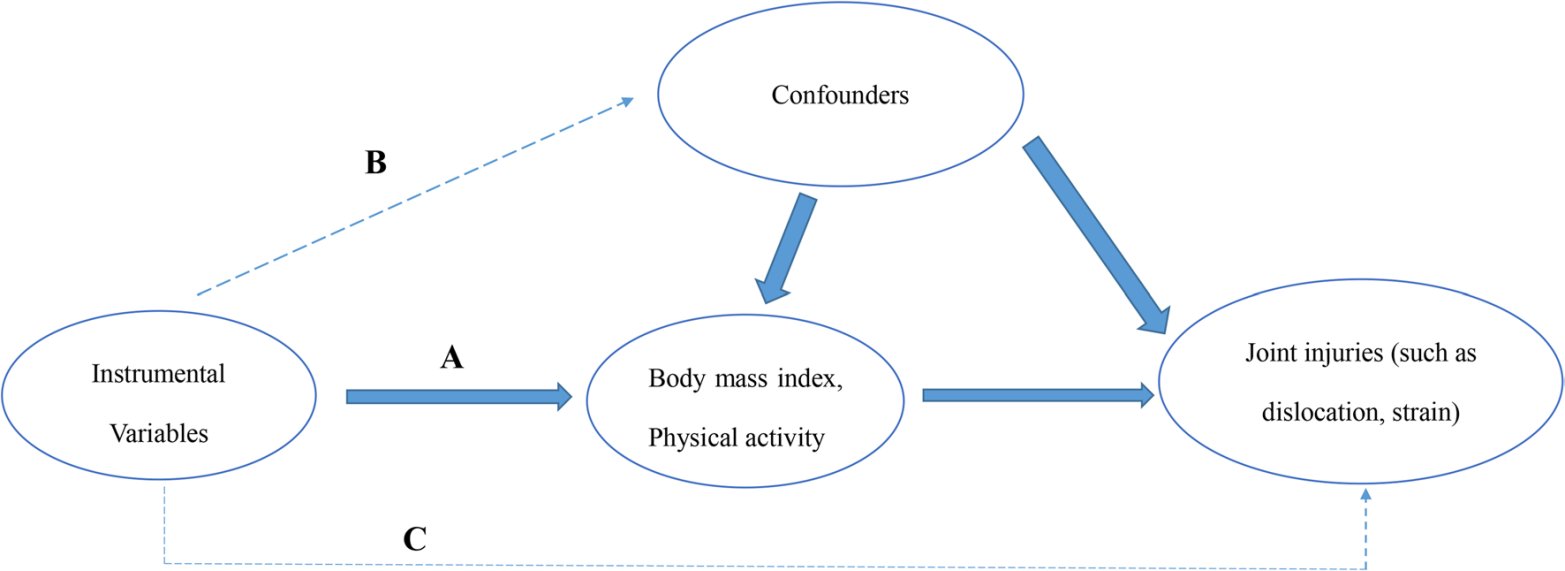
Three basic presumptions of the MR study. **A** The IVs must be correlated with the exposures (BMI or PA). **B** IVs must be entirely unconnected with confounders, and **C** IVs should not be directly linked to the outcomes (joint sports injuries). *MR* Mendelian randomization, *IVs* instrumental variables, *BMI* body mass index, *PA* physical activity

This research employed a two-sample MR analysis to explore the causal effect of BMI on joint sports injuries. Figure 1 presents a graphical diagram of the study design strategy; that is, the analysis process must meet three basic requirements: (A) IVs must be robustly linked to the exposure (BMI, PA); (B) IVs are supposed to be isolated from any potential confounders; and (C) SNPs must be linked to the risk of outcomes (joint sports injuries) only via exposure (BMI, PA). All datasets utilized for analysis are summary-level GWAS and publicly available. Ethical approvals and informed consent are also fully qualified by their corresponding institutions. Ultimately, this research report complies with the STROBE-MR guideline, which is beneficial for peer evaluation and result interpretation [15].

### Data sources and selection of IVs

For exploring the causal relationship between BMI and joint injuries, data available for adult BMI was acquired from the currently largest GWAS for BMI from the Genetic Investigation of Anthropometric Traits (GIANT) consortium (https://portals.broadinstitute.org/collaboration/giant/index.php/GIANT_consortium), which contains samples of 681,275 European populations [16]. Outcome data for various body parts of sports injuries, such as dislocation, sprain, and strain, were extracted from the FinnGen [17], which can be easily accessed via the IEU Open GWAS Project (https://gwas.mrcieu.ac.uk/). Further details regarding the exposure and outcomes were provided in Additional file 1: Table S1.

We selected two sets of BMI-associated IVs: one contained 656 primary genome-wide significant (*p* < 5 × 10^−8^) genetic variants, and the other group contained 77 IVs that did not overlap with the first one based on previously published studies [16, 18] (Additional file 1: Tables S2 and S3).

A large GWAS involving 377,234 individuals from the UK Biobank yielded exposure data on physical activities [19]. In their research, an accelerometer worn on the wrist or a questionnaire was employed for estimating PA intensity [20]. Four phenotypes of PA from their study were investigated, including moderate-to-vigorous physical activity (MVPA), vigorous physical activity (VPA), accelerometer-based physical activity measurement (overall average acceleration) (AccAve), and 2–3 days/week or more in doing strenuous sports or other exercises for 15–30 min or greater (SSOE) [19]. Detailed information such as overall sample size, resource link, and number of SNPs was exhibited in Additional file 1: Table S1.

Then, these IVs associated with four PA phenotypes at genome-wide significance (*p* < 5E−08) throughout the genome were clumped and harmonized in R (version 4.2.1) utilizing the TwoSampleMR package (version 0.5.6) [21]. SNPs were removed during analysis when their linkage disequilibrium (LD) was consistent with *r*^2^ > 0.01 and clumping distance < 10,000. Beyond that, proxy SNPs serve as substitutes for those SNPs that are palindromic with intermediate allele frequencies [22]. To avoid weak instrumental bias, the *F* statistic was used to determine the strength of each IV. If *F* > 10, it can be considered that the association between IVs and exposure is effective, and the MR results are not affected by weak instrumental bias [23]. Finally, details of the SNPs selected as IVs for BMI and PA were provided in Additional file 1: Tables S2–S3, S16, respectively.

### MR analysis

Following harmonization of the effect alleles across the GWAS data of BMI, PA, and joint injuries, we conducted five methods of MR analysis to identify the causal effect of BMI and PA on joint injuries, which are inverse variance weight (IVW), weighted median, MR-Egger, simple mode, and weighted mode. The IVW was considered the main outcome because this method assumes that IVs affect the outcome only via exposure and not in any other way [24]. Moreover, the MR-Egger and weighted median methods provide more modest estimated values under a more permissive assumption but with low precision (broader CIs). In this study, it was considered that there was a causal effect when all those MR approaches were consistent in direction. To address multiple testing, a Bonferroni-corrected *p* value of 0.00384 (i.e., 0.05/13 putative outcomes) was regarded as significant, and *p* values between 0.00384 and 0.05 were defined as nominal significance.

### Sensitivity analysis

Complying with the MR study design strategy, the IVs affect outcomes only via exposure. The estimated values may be inaccurate if the SNPs used as IVs have horizontal pleiotropy. To detect this, the intercept contained in the MR-Egger method must be markedly different from 0, as well as the visual observation of the funnel plot, in which asymmetry represents horizontal pleiotropy [24, 25]. Simultaneously, the MR Pleiotropy Residual Sum and Outlier (MR-PRESSO) method was also conducted to check and rectify horizontal pleiotropy [26]. Eventually, Cochran’s *Q* test was implemented to determine whether there was heterogeneity in each IV [27].

## Results

### Genetically predicted increased BMI and joint sports injuries

**Fig. 2:**
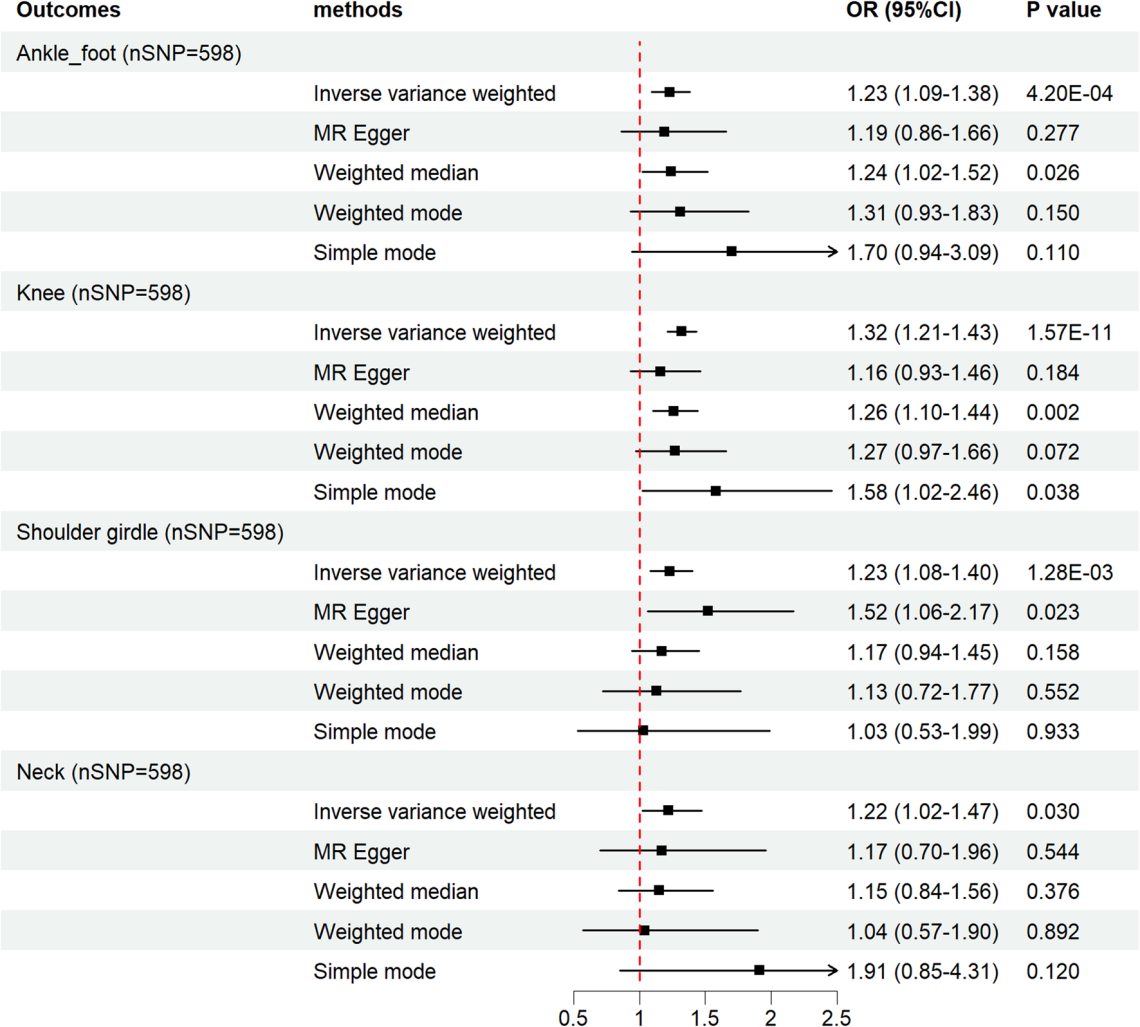
A forest plot depicting the results of five different MR estimating methods on the associations between BMI (IVs derived from the Yengo et al. study) and the joint sports injury risk of the ankle–foot, knee, shoulder girdle, and neck. *MR* Mendelian randomization, *IVs* instrumental variables, *BMI* body mass index

Additional file 1: Table S13 exhibited the selected IVs strongly associated with an increased BMI, which were derived from the recent study by Yengo et al. [16]. Results showed that increased BMI was significantly correlated with dislocation, sprain, and strain of joints and ligaments (called together “sports injury,” the same below) at ankle–foot level (IVW: OR 1.23, 95% CI 1.09–1.37, *p* = 4.20E−04), the knee (IVW: OR 1.32, 95% CI 1.21–1.43, *p* = 1.57E−11), and shoulder girdle (IVW: OR 1.23, 95% CI 1.08–1.40, *p* = 1.28E−03). Additionally, a nominal significance was revealed after analyzing the correlation between BMI and sports injury at the neck level (IVW: OR 1.22, 95% CI 1.02–1.47, *p* = 0.030) (Fig. 2). Detailed information on each MR analysis was provided in Additional file 1: Tables S4–S7. No evidence was found of directional pleiotropy through the MR-Egger intercept scatter plot or the funnel plot (Additional file 2: Figs. S1–S4). In the MR-PRESSO global test, *p* = 0.021, when analyzing the association between BMI and ankle injury, two SNPs were identified as outliers (rs2230590 and rs12364470). MR-PRESSO outlier-corrected estimates stayed in accordance with the original analysis (Additional file 1: Table S14).

**Fig. 3:**
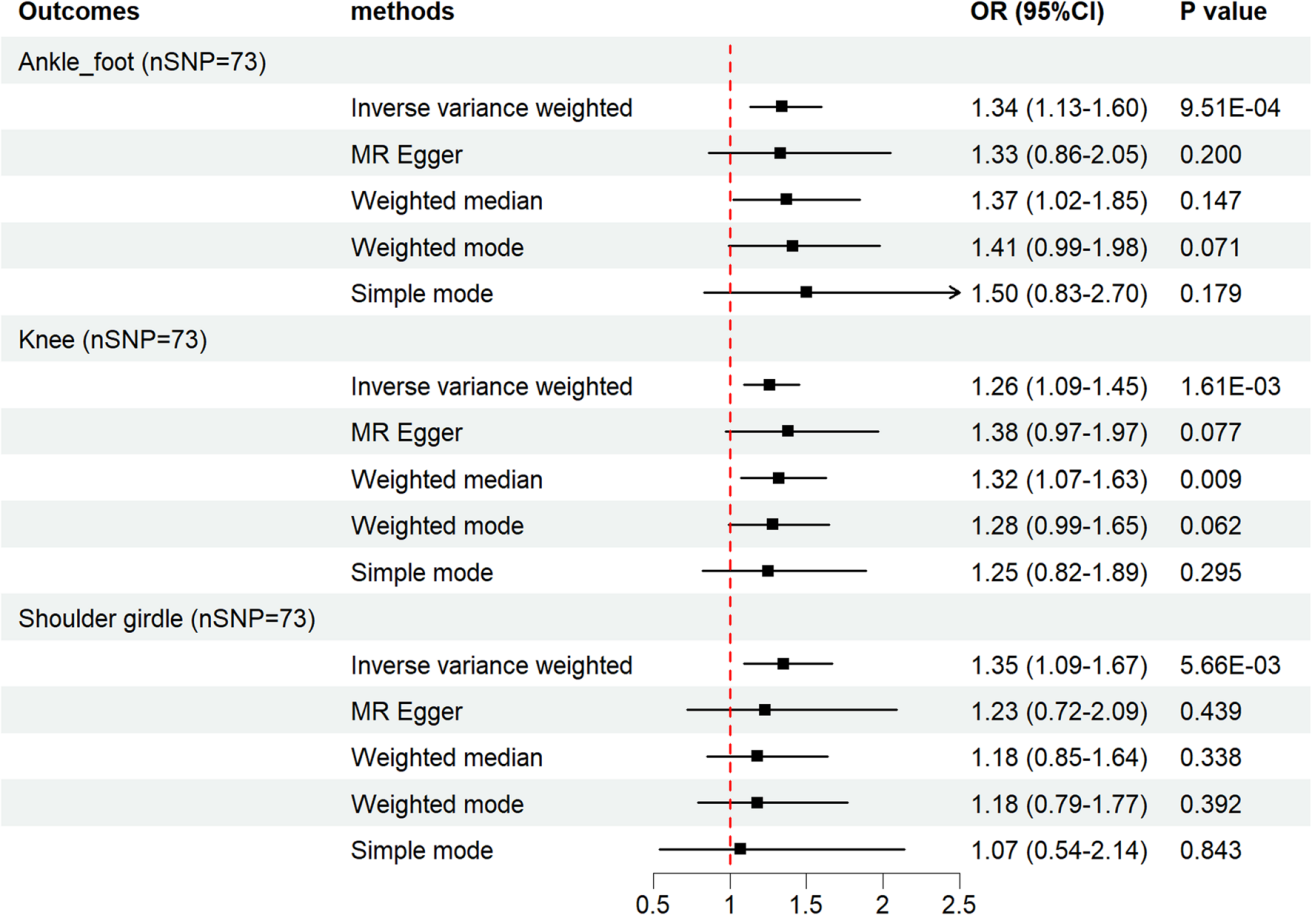
A forest plot depicting the results of five different MR estimating methods on the associations between BMI (IVs derived from the Locke et al. study) and the joint sports injury risk of the ankle–foot, knee, and shoulder girdle. *MR* Mendelian randomization, *IVs* instrumental variables, *BMI* body mass index

For further confirmation of the results, another BMI GWAS obtained from the study by Locke et al. [18] was implemented to validate the causal effect of BMI on the risk of joint injuries. Significant effects were revealed for increased BMI on sports injury risk of ankle–foot (IVW: OR 1.34, 95% CI 1.13–1.60, *p* = 9.51E−04), knee (IVW: OR 1.26, 95% CI 1.09–1.45, *p* = 1.63E−03). Additionally, a nominal significant causal effect was identified between BMI and shoulder girdle injury (IVW: OR 1.35, 95% CI 1.09–1.67, *p* = 5.66E−03) (Fig. 3, Additional file 1: Table S15). Detailed information on each MR analysis was exhibited in Additional file 1: Tables S8–S10. Furthermore, sensitivity analysis suggested no heterogeneity or horizontal pleiotropy through Cochran’s Q test and the MR-Egger intercept. Meanwhile, the MR-PRESSO test revealed no potential outliers (Additional file 1: Table S15). The leave-one-out results indicated that the causal effect was not actuated by a single IV (Additional file 3: Figs. S5–S7).

### Genetically predicted physical activities and joint sports injuries

**Fig. 4:**
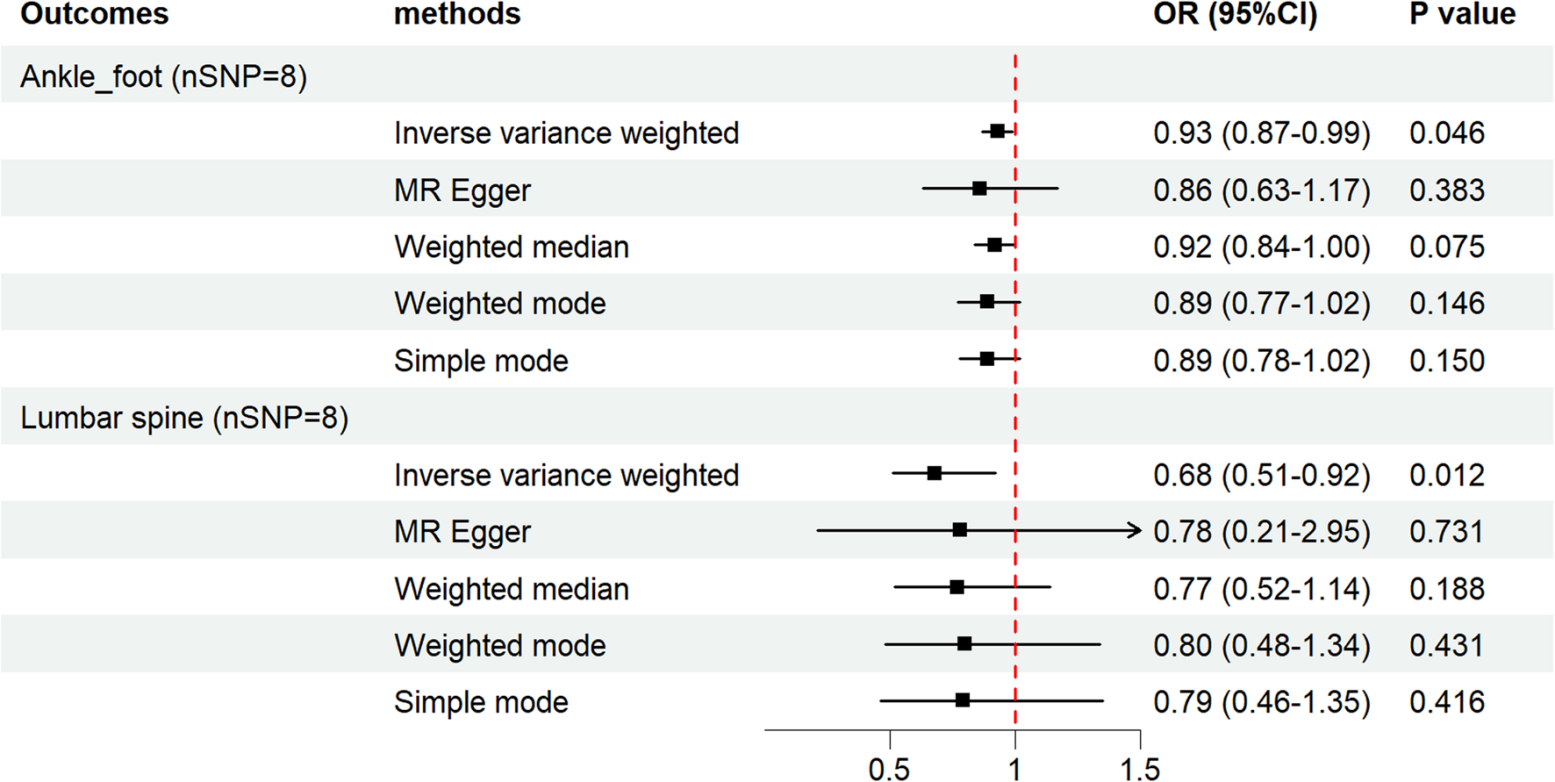
A forest plot exhibiting the results of five MR estimating methods on the correlations between AccAve and the joint sports injury risk of the ankle–foot and lumbar spine. *MR* Mendelian randomization, *AccAve* accelerometer-based physical activity measurement (average acceleration)

Additional file 1: Table S16 displayed the IVs we used in analyzing the causal effect of four kinds of physical activities on joint sports injuries. No causal associations were observed when analyzing the effect of physical activities on joint sports injuries except for accelerometer-based physical activity measurement (average acceleration) (AccAve) (Additional file 1: Tables S17–S20). Evidence of a protective causal correlation was discovered between AccAve and injury at the ankle and foot level (IVW: OR 0.93, 95% CI 0.87–0.99, *p* = 0.046) and injury of the lumbar spine (IVW: OR 0.68, 95% CI 0.51–0.92, *p* = 0.012) (Fig. 4). Information on MR analyses between AccAve and sports injuries at the ankle–foot and lumbar spine was provided in Additional file 1: Tables S11–S12. Sensitive analysis revealed that no heterogeneity or horizontal pleiotropy existed in this analysis via Cochran’s *Q* test, the egger intercept, MR-PRESSO (Additional file 1: Table S17), as well as the scatter plot, funnel plot, and forest plot of the leave-one-out analysis (Additional file 4: Figs. S8–S9).

### Effect of genetically predicted BMI on the risk of injury at ankle and foot level by adjusting physical activity

**Fig. 5:**
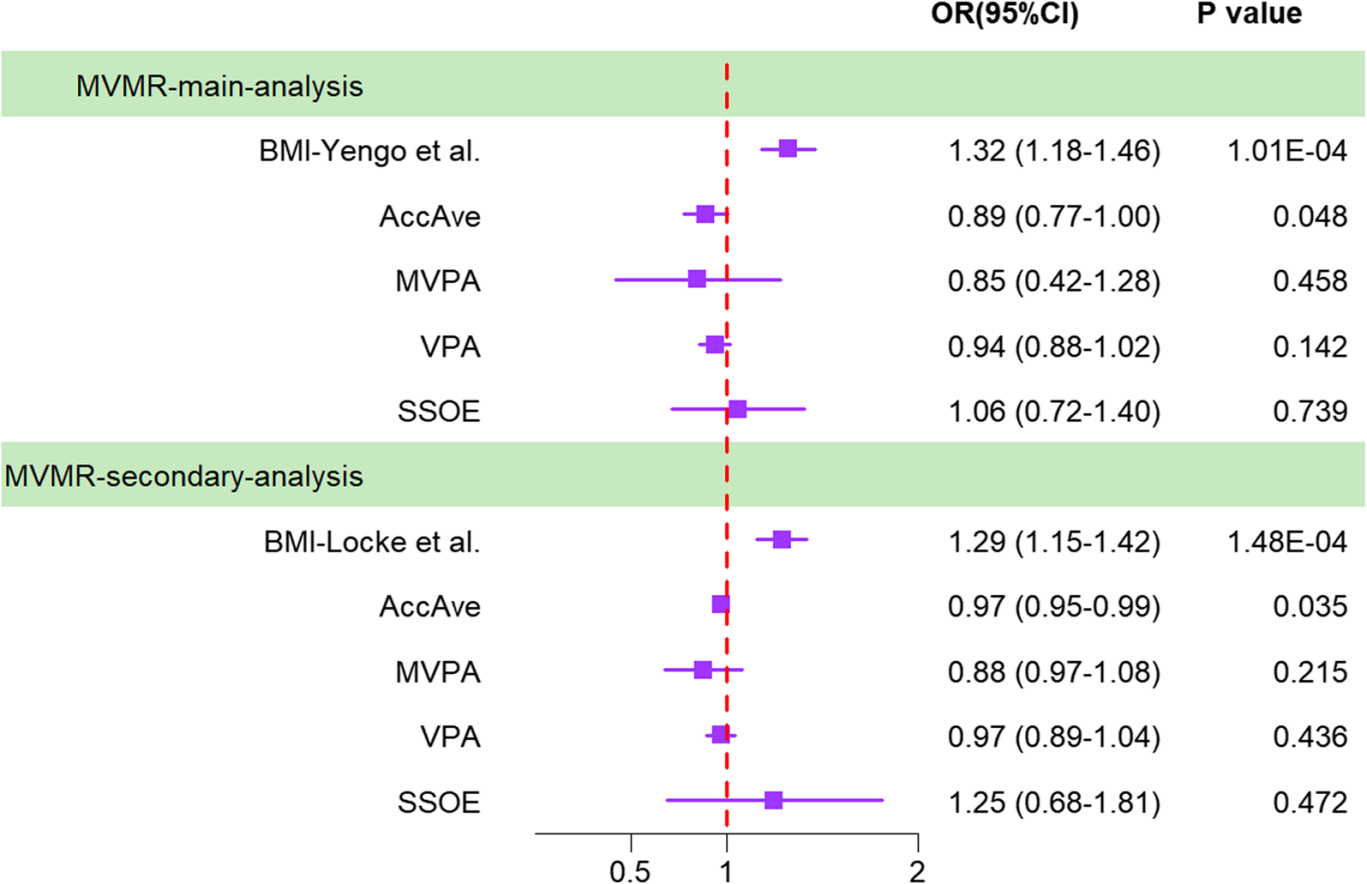
Forest plot of MVMR estimates from the IVW method of BMI and PA with the joint sports injury risk of the ankle–foot. *MVMR* multivariate Mendelian randomization, *IVW* inverse-variance weighted, *BMI* body mass index, *PA* physical activity, *AccAve* accelerometer-based physical activity measurement (average acceleration), *MVPA* moderate-to-vigorous physical activity, *VPA* vigorous physical activity, *SSOE* strenuous sports or other exercises Accelerometer-based PA (acceleration fraction > 425 mg)

Ultimately, the PhenoScanner online website tool (http://www.phenoscanner.medschl.cam.ac.uk/) was conducted to confirm whether the SNPs served as IVs in connection with other phenotypes. Several SNPs related to BMI were reported to have an impact on the risk of injury at the ankle and foot level [28, 29] (Additional file 1: Table S21). Accordingly, multivariate MR (MVMR) was implemented to illustrate the causal associations between physical activity and the risk of injury at the ankle and foot level, adjusting potential pleiotropy related to BMI. The BMI summary data are exhibited in Additional file 1: Table S1. Results showed that the effect of AccAve on the risk of injury at the ankle–foot still had a nominal statistical significance after adjusting BMI (Fig. 5).

## Discussion

The previous studies provided evidence that participation in certain sports and BMI were risk factors that heightened the risk of suffering joint sports injuries such as sprains and strains. However, such conclusions remained debatable across studies. In the meantime, uncertain confounding factors in observational research might be influential on the correlation results. Studies on the epidemiology of sports joint injuries indicated that increased BMI and greater physical activity were risk factors for sports joint injuries [12, 30–33]. Nevertheless, the sample size in these studies was insufficient, and only BMI or a single physical activity was analyzed without correcting for potential bias. In this study, we systematically explored the causal effect of BMI and different intensities of physical activity on 13 types of joint injuries in sports by employing two-sample and MVMR methods. Significant positive causal correlations were identified between BMI and sports injury risk of the ankle, knee, and shoulder girdle. Further investigation showed that AccAve was negatively correlated with the injury risk of the ankle and lumbar spine. These findings offered a genetic perspective on the causal relationships between BMI, physical activity, and the risk of joint sports injury, which might have clinical value for clinicians and patients.

Increased body mass index was reported to be a significant risk factor for knee, shoulder, and ankle–foot injuries, including meniscal tears, rotator cuff disease, plantar fasciitis, and ankle sprains [12, 32–34]. Despite almost all epidemiological investigations supporting a higher BMI as a risk factor for joint sports injuries, the mechanism behind it is still controversial. One hypothesis holds that a strong mass moment of inertia acting around the ankle leads to ankle sprains and other lower extremity injuries [35, 36]. Other theories believe BMI is a valid risk factor as obesity is connected with chronic inflammation [37], which may contribute to degenerated tendons and pain. Obesity is also linked to other diseases like dyslipidemia and high blood pressure [38], which may also raise the risk of shoulder injury [39, 40]. Using the MR method, we identified increased BMI as a risk factor for injuries to the ankle–foot, knee, and shoulder. Further, overall average acceleration (AccAve) was demonstrated as a protective factor against injury to the ankle–foot and lumbar spine. Previous studies suggested men who had better scores in push-ups and sit-ups (number of push-ups or sit-ups completed in 2 min) and better performance in a 2-mile run had a higher incidence of ankle sprains than their counterparts [12]. Basketball, football, and soccer account for over half of all ankle sprains sustained while participating in physical activity, which accounts for almost half of all ankle sprains [31]. Ankle sprains are thought to occur more likely in physical activities involving frequent contact with others, as well as repeated running, jumping, and sharp cutting actions that subject the ankle to greater angular and rotational pressure [13, 41–43]. Compared to lower extremity injuries, the incidence of lumbar spine injuries was uncommon [44]. Despite this, lumbar spine injuries are still a non-negligible reason athletes are absent from competition [45]. Moreover, studies found that athletic competition is more likely to result in a lumbar spine injury than routine training [44], which proved that injuries are more likely to be caused by enhanced athletic effort in sports competitions [46]. In contrast to these perspectives, our results suggested a protective role for overall average acceleration (AccAve) against ankle–foot and lumbar spine injuries. Although the precise mechanism of this protection is yet unidentified, one possible interpretation is that moderate physical activity, such as slow walking, can strengthen multi-muscular coordination for smooth motion, like neuromuscular training [47, 48]. It is worth noting that vigorous physical activity has no causal relationship with the risk of ankle–foot and lumbar spine injuries, according to the current results. The inconsistency between the results and the previous epidemiologic study may be because the definition of vigorous physical activity in the original GWAS data needed to be clarified. People were asked to answer a questionnaire or wear an accelerometer; fraction accelerations greater than 425 milligravities (approximately 4.2 m/s^2^) were considered high intensity [19], which may be affected by cognitive bias or not reach the condition of causing joint injuries.

However, the study is subject to some limitations. Despite the statistical significance of the results, they do not indicate clinical relevance. The case number of some sports injury sites is still small; more clinical traits and epidemiological research with larger sample sizes are urgently needed to confirm the conclusions. Second, self-reported measures of physical activity may be influenced by mental states and cognitive bias. Even though this does not weaken the reliability of self-reported evaluations [49], objective evaluations are still needed to confirm their results. Meanwhile, accelerometer-based assessments of physical activity have their limitations. Movement posture, intensity of muscles and ligaments, and nonambulatory activity (e.g., bicycle) are difficult to record. Therefore, further investigation is required into the causal links between more classified physical activities and joint injuries. Third, the outcome data extracted from the FinnGen database concerning different joint injuries need to be updated due to insufficient cases.

In summary, this research conducted a genetic approach and evidently found that BMI is causally connected with the injury risk of the ankle–foot, knee, and shoulder. Additionally, overall average acceleration (AccAve) could causally decrease the injury risk of the ankle–foot and lumbar spine. These conclusions supported a genetic perspective about the causal relationships between BMI, physical activity, and the risk of joint sports injury and offered clinical advice for patients and caregivers.

### Supplementary Information

**Additional file 1. **Supplementary Tables S1 to S21.

**Additional file 2. **Supplementary Figures 1 to 4.

**Additional file 3.  **Supplementary Figures 5 to 7.

**Additional file 4. **Supplementary Figures 8 to 9.

## Data Availability

The datasets presented in this study can be found in online repositories. The names of the repository/repositories and accession number(s) can be found in the article/Additional file 1.
