# Supplementary material for: Causal effect of body mass index and physical activity on the risk of joint sports injuries: Mendelian randomization analysis in the European population
Source: J Orthop Surg Res. 2023 Sep 12;18:676. doi: 10.1186/s13018-023-04172-y (PMC10496185; doi:10.1186/s13018-023-04172-y)
Supplement: Supplementary file 2 — Additional file 2. Supplementary Figures 1 to 4. [file 13018_2023_4172_MOESM2_ESM.pdf]

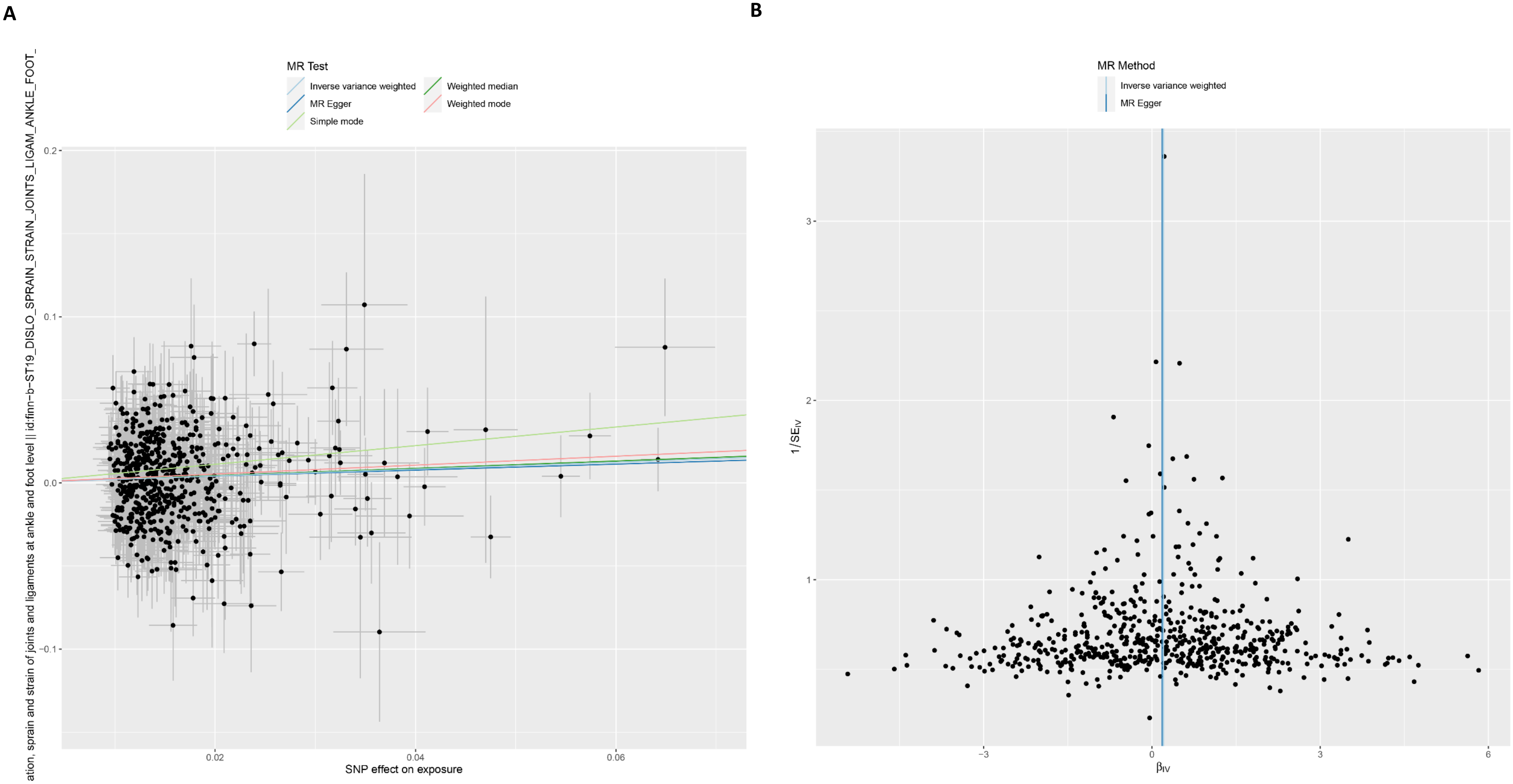

Supplementary Figure 1 Scatter plot and funnel plot of the relation between BMI (genetic variants derived from Yengo et al study PMID 30124842) and risk of ankle joint injury, Yengo et al study (PMID 30124842). **(A)** Scatter plot of SNP potential effects on BMI and injury at ankle and foot level **(B)**. Funnel plot exhibiting the estimation using the inverse of the standard error of the casual estimate with each individual SNP as a tool. Abbreviation: SNP, single nucleotide polymorphism; BMI, body mass index; IVs, instrumental variables

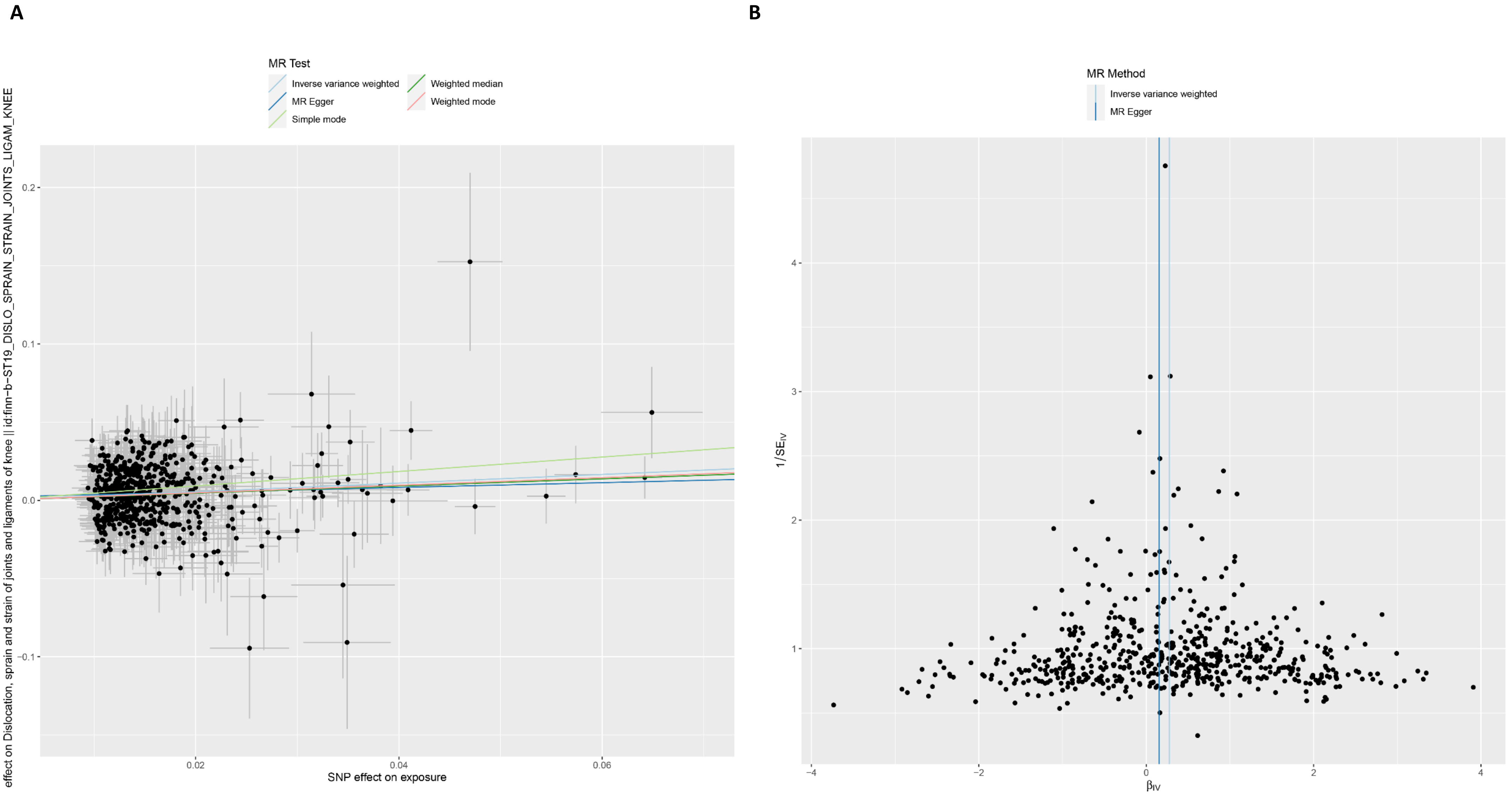

Supplementary Figure 2 Scatter plot and funnel plot of the relation between BMI (genetic variants derived from Yengo et al study PMID 30124842) and risk of knee joint injury, Yengo et al study (PMID 30124842). **(A)** Scatter plot of SNP potential effects on BMI and knee injury **(B)**. Funnel plot exhibiting the estimation using the inverse of the standard error of the casual estimate with each individual SNP as a tool. Abbreviation: SNP, single nucleotide polymorphism; BMI, body mass index; IVs, instrumental variables

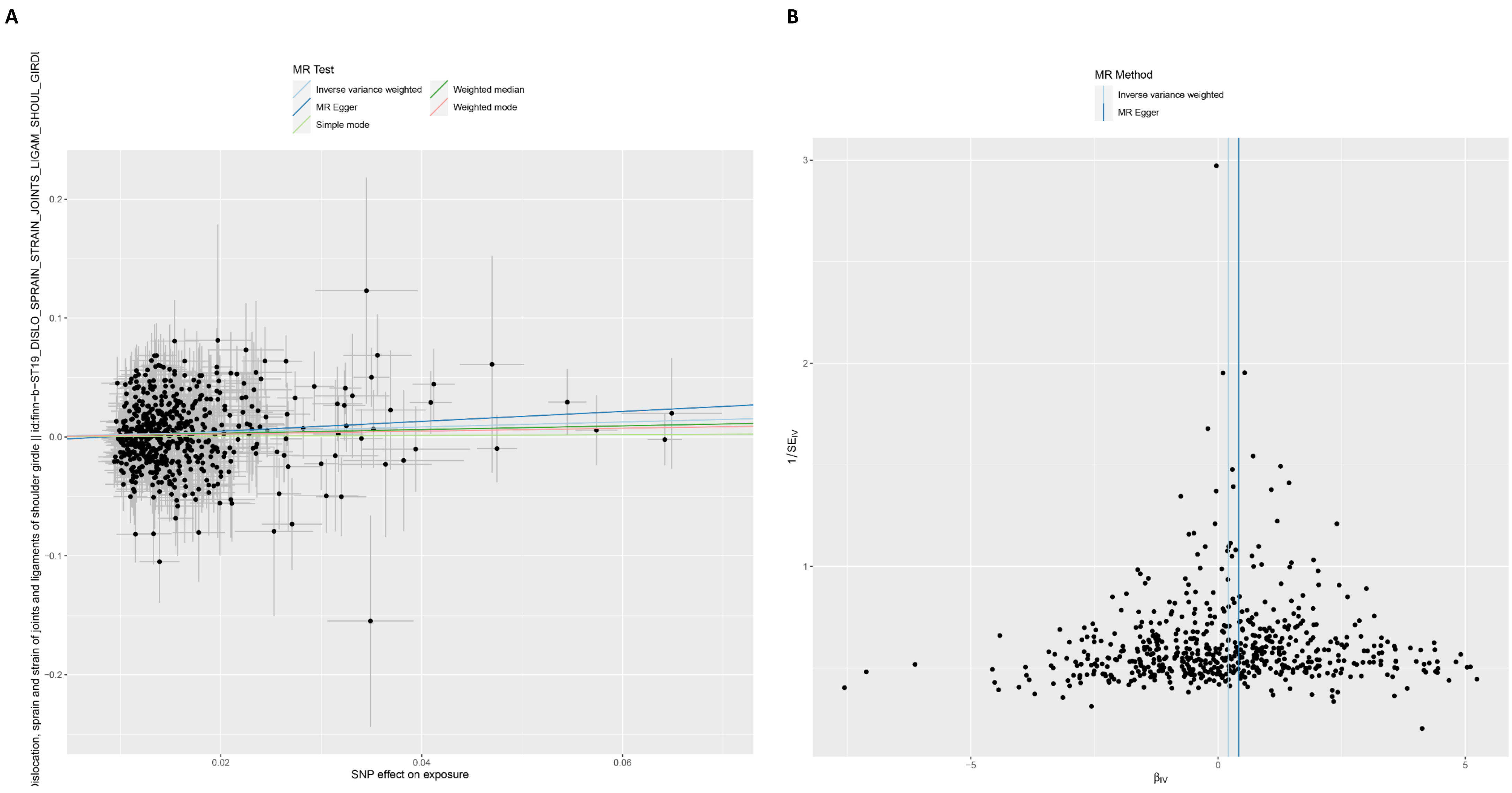

Supplementary Figure 3 Scatter plot and funnel plot of the relation between BMI (genetic variants derived from Yengo et al study PMID 30124842) and risk of shoulder girdle injury, Yengo et al study (PMID 30124842). **(A)** Scatter plot of SNP potential effects on BMI and shoulder girdle injury **(B)**. Funnel plot exhibiting the estimation using the inverse of the standard error of the casual estimate with each individual SNP as a tool. Abbreviation: SNP, single nucleotide polymorphism; BMI, body mass index; IVs, instrumental variables

A

B

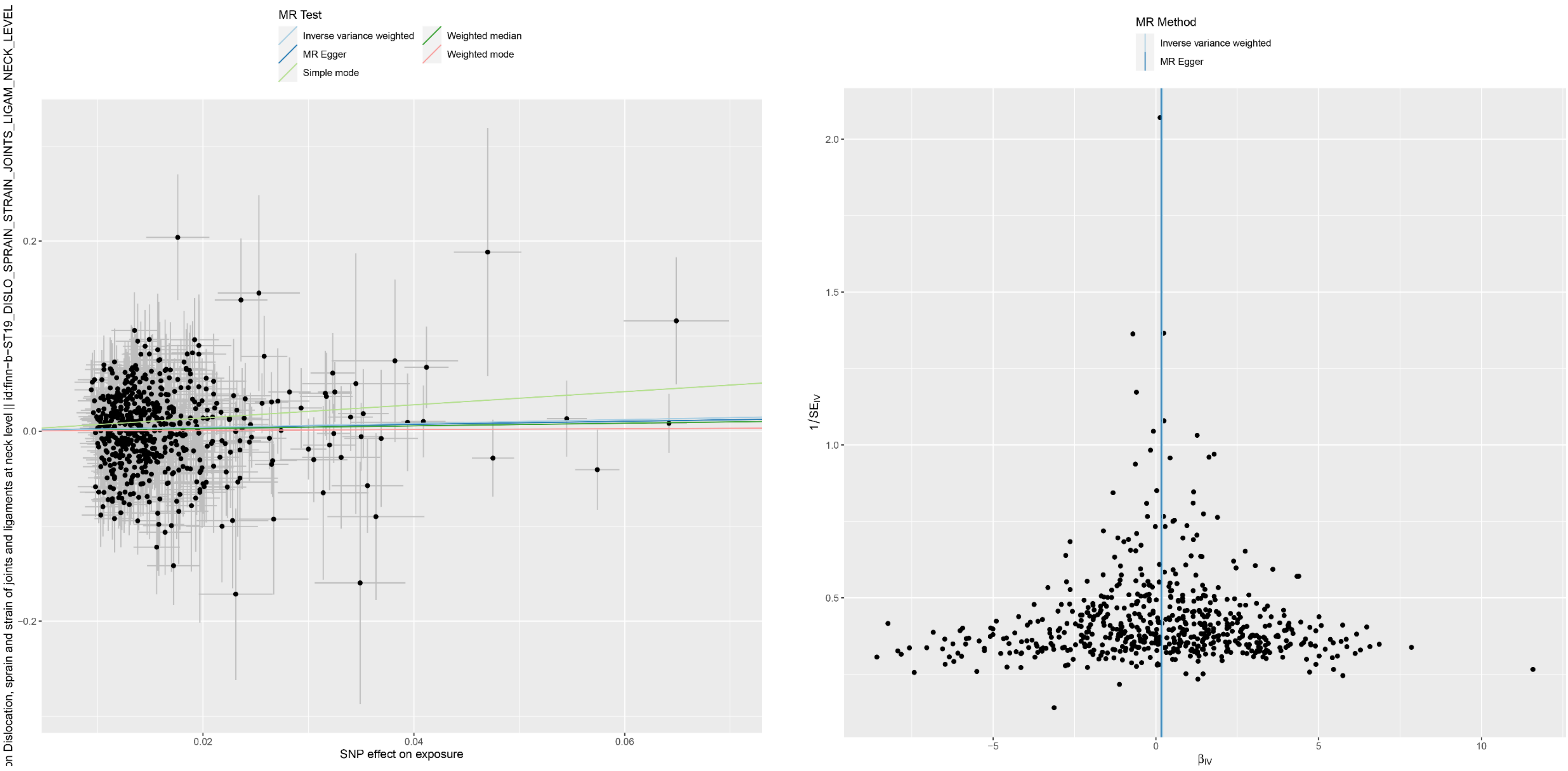

Supplementary Figure 4 Scatter plot and funnel plot of the relation between BMI (genetic variants derived from Yengo et al study PMID 30124842) and risk of neck injury, Yengo et al study (PMID 30124842). **(A)** Scatter plot of SNP potential effects on BMI and neck injury **(B)**. Funnel plot exhibiting the estimation using the inverse of the standard error of the casual estimate with each individual SNP as a tool. Abbreviation: SNP, single nucleotide polymorphism; BMI, body mass index; IVs, instrumental variables
