# Supplementary material for: Causal effect of body mass index and physical activity on the risk of joint sports injuries: Mendelian randomization analysis in the European population
Source: J Orthop Surg Res. 2023 Sep 12;18:676. doi: 10.1186/s13018-023-04172-y (PMC10496185; doi:10.1186/s13018-023-04172-y)
Supplement: Supplementary file 3 — Additional file 3. Supplementary Figures 5 to 7. [file 13018_2023_4172_MOESM3_ESM.pdf]

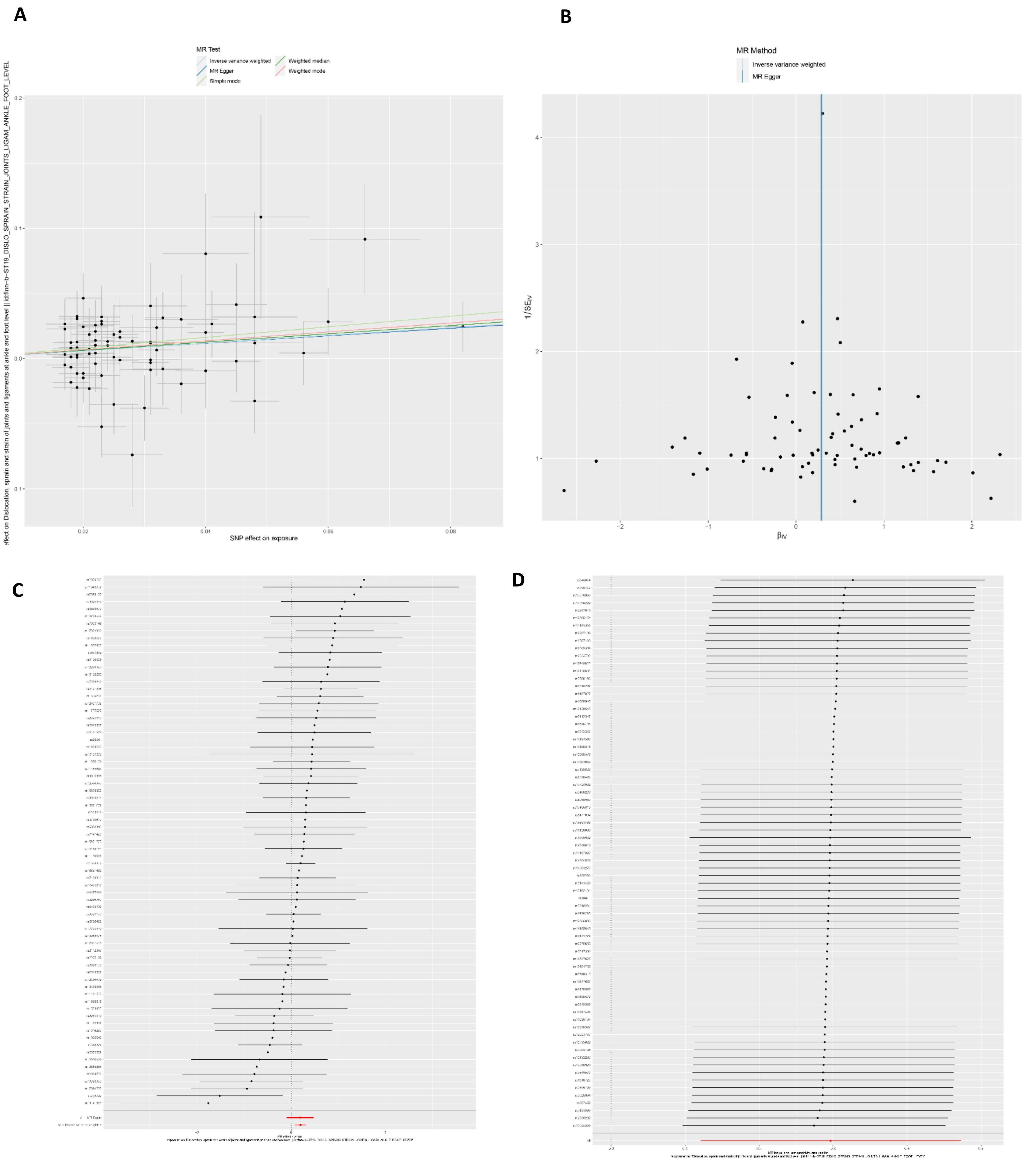

Supplementary Figure 5 Mendelian analysis results for BMI (genetic variants derived from Locke et al. study, PMID 25673413) on the risk of ankle and foot injuries. **(A)** Scatter plot of SNP potential effects on BMI and injury at ankle and foot level (**B**). Funnel plot exhibiting the estimation using the inverse of the standard error of the casual estimate with each individual SNP as a tool. (**C**) Forest plot of the casual effect of BMI on the risk of injury at ankle and foot level (**D**) Forest plot of the leave-one-out sensitivity analysis, where each SNP was iteratively from the IVs. Abbreviation: SNP, single nucleotide polymorphism; BMI, body mass index; IVs, instrumental variables

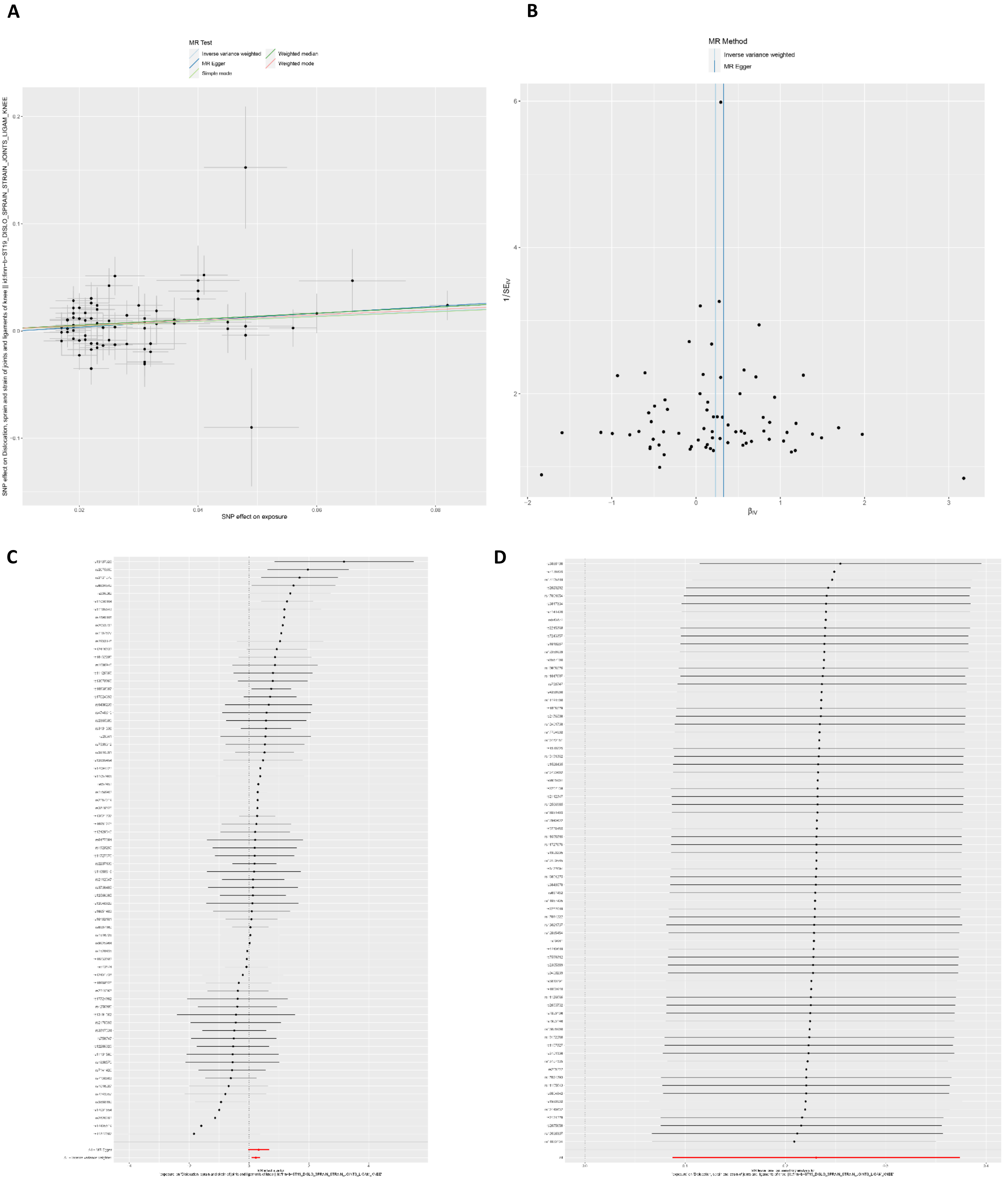

Supplementary Figure 6 Mendelian analysis results for BMI (genetic variants derived from Locke et al. study, PMID 25673413) on the risk of knee injury. **(A)** Scatter plot of SNP potential effects on BMI and knee injury (**B**). Funnel plot exhibiting the estimation using the inverse of the standard error of the casual estimate with each individual SNP as a tool. (**C**) Forest plot of the casual effect of BMI on the risk of knee injury (**D**) Forest plot of the leave-one-out sensitivity analysis, where each SNP was iteratively from the IVs. Abbreviation: SNP, single nucleotide polymorphism; BMI, body mass index; IVs, instrumental variables

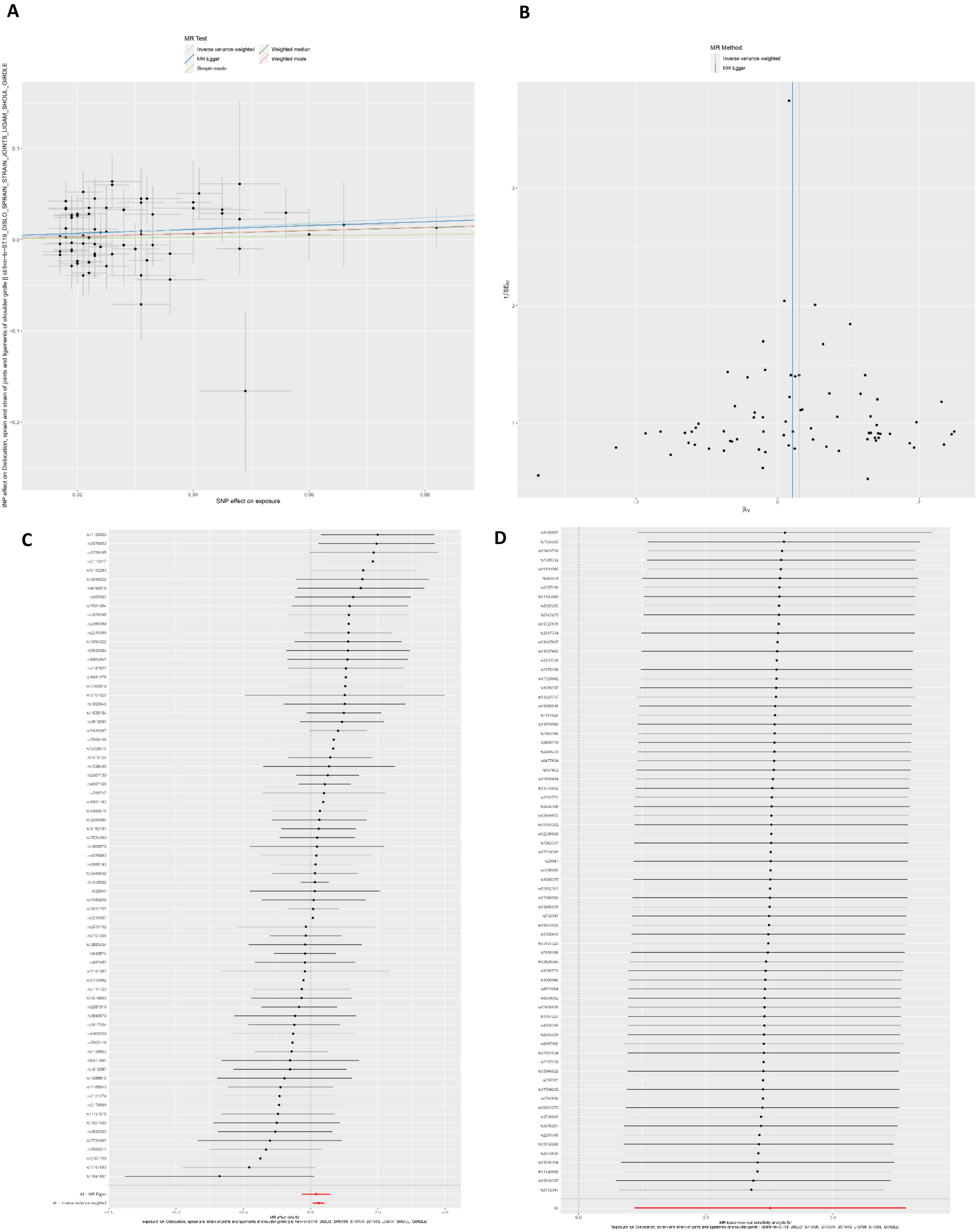

Supplementary Figure 7 Mendelian analysis results for BMI (genetic variants derived from Locke et al. study, PMID 25673413) on the risk of shoulder girdle injury. **(A)** Scatter plot of SNP potential effects on BMI and knee injury (**B**). Funnel plot exhibiting the estimation using the inverse of the standard error of the casual estimate with each individual SNP as a tool. (**C**) Forest plot of the casual effect of BMI on the risk of knee injury (**D**) Forest plot of the leave-one-out sensitivity analysis, where each SNP was iteratively from the IVs. Abbreviation: SNP, single nucleotide polymorphism; BMI, body mass index; IVs, instrumental variables
