# Supplementary material for: Causal effect of body mass index and physical activity on the risk of joint sports injuries: Mendelian randomization analysis in the European population
Source: J Orthop Surg Res. 2023 Sep 12;18:676. doi: 10.1186/s13018-023-04172-y (PMC10496185; doi:10.1186/s13018-023-04172-y)
Supplement: Supplementary file 4 — Additional file 4. Supplementary Figures 8 to 9. [file 13018_2023_4172_MOESM4_ESM.pdf]

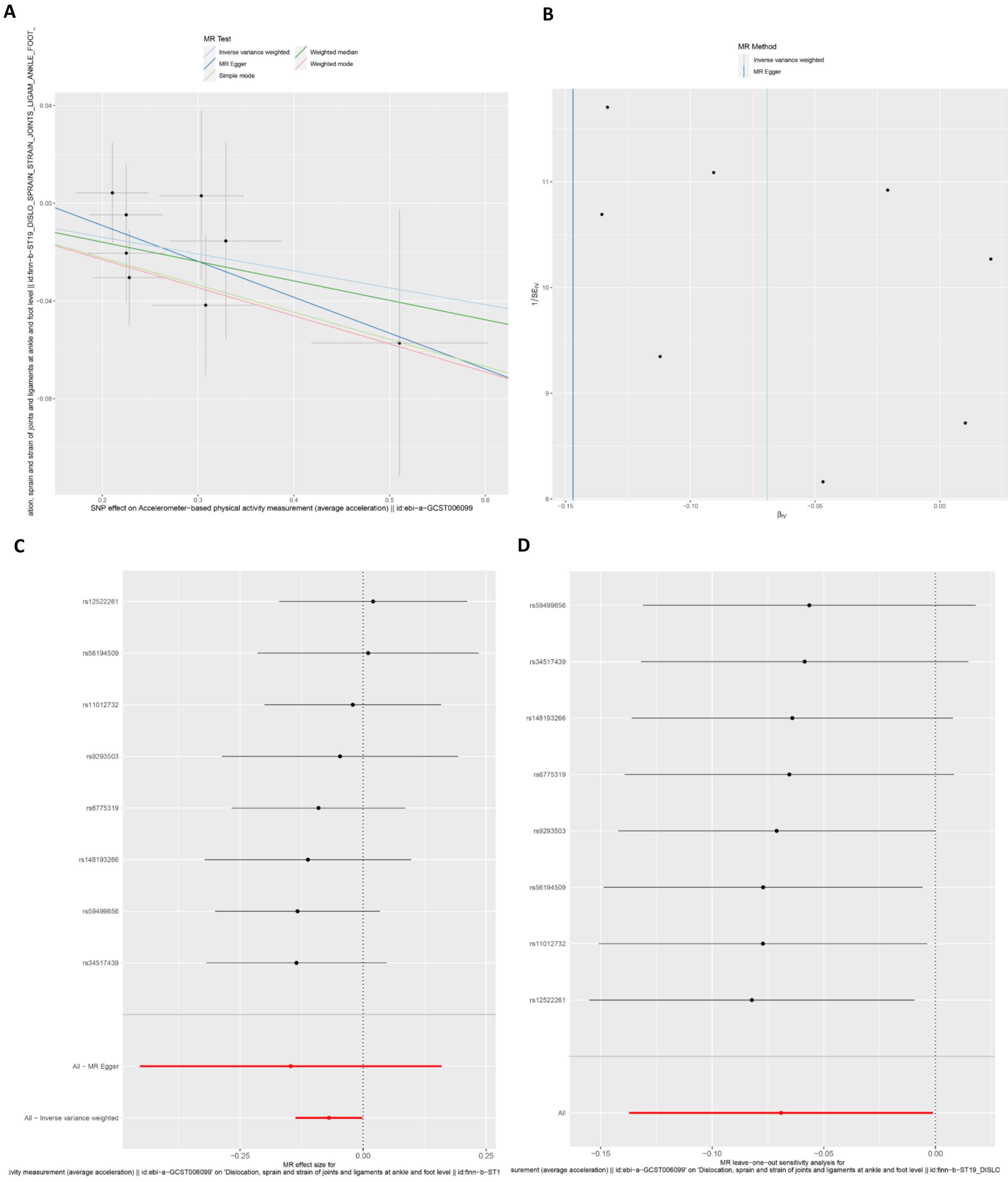

Supplementary Figure 8 Mendelian analysis results for AccAve on the risk of ankle and foot injuries. **(A)** Scatter plot of SNP potential effects on AccAve and injury at ankle and foot level (**B**). Funnel plot exhibiting the estimation using the inverse of the standard error of the casual estimate with each individual SNP as a tool. (**C**) Forest plot of the casual effect of AccAve on the risk of injury at ankle and foot level (**D**) Forest plot of the leave-one-out sensitivity analysis, where each SNP was iteratively from the IVs. Abbreviation: SNP, single nucleotide polymorphism; AccAve, Accelerometer-based physical activity measurement (average acceleration); IVs, instrumental variables

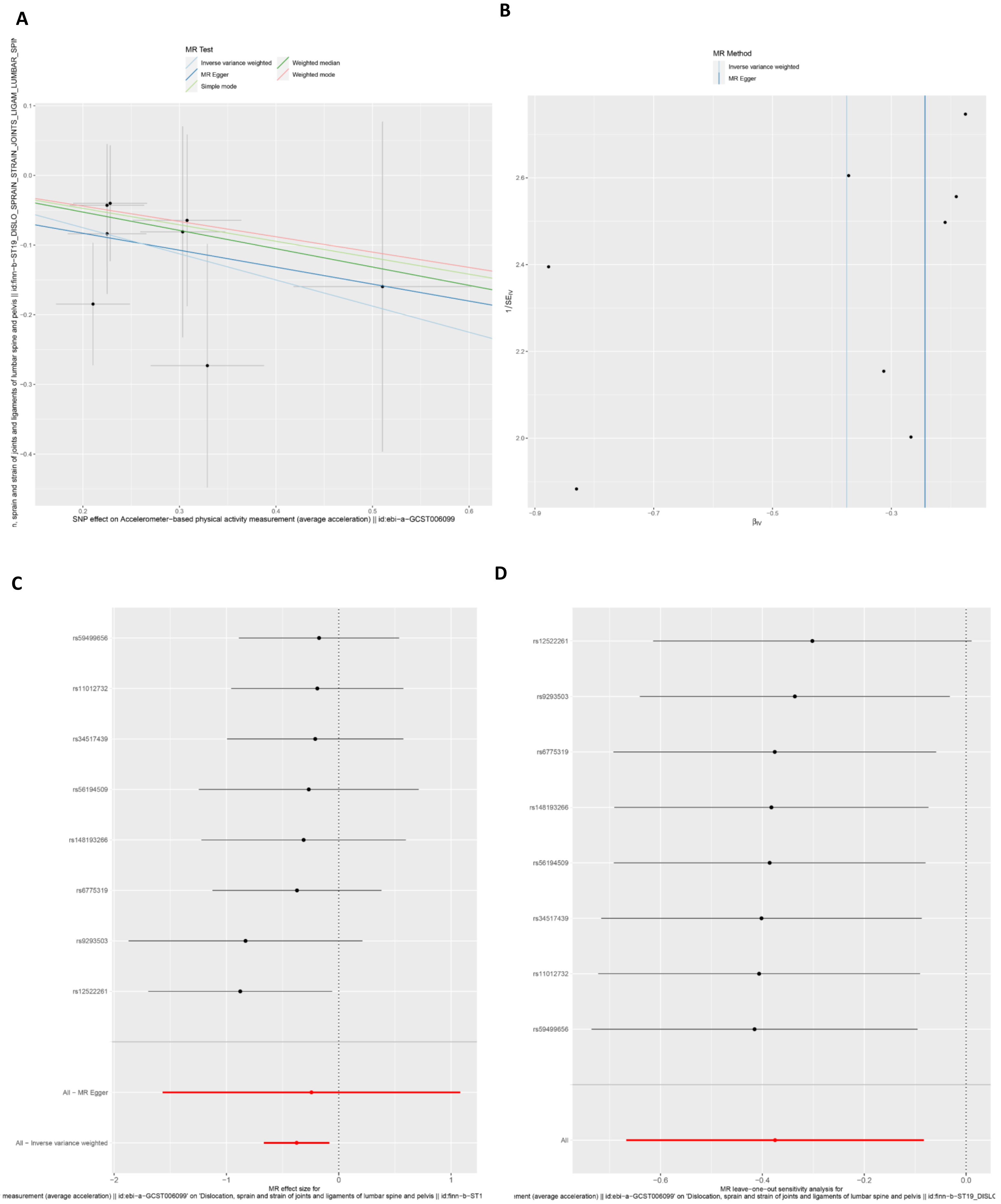

Supplementary Figure 9 Mendelian analysis results for AccAve on the risk of lumbar spine and pelvis injuries. **(A)** Scatter plot of SNP potential effects on AccAve and the risk of lumbar spine and pelvis injury (**B**). Funnel plot exhibiting the estimation using the inverse of the standard error of the causal estimate with each individual SNP as a tool. (**C**) Forest plot of the causal effect of AccAve on the risk of injury at ankle and foot level (**D**) Forest plot of the leave-one-out sensitivity analysis, where each SNP was iteratively from the IVs. Abbreviation: SNP, single nucleotide polymorphism; AccAve, Accelerometer-based physical activity measurement (average acceleration); IVs, instrumental variables
